# Ethical and practical considerations concerning perimortem sperm procurement in a severe neurologically damaged patient and the apparent discrepancy in validation of proxy consent in various postmortem procedures

**DOI:** 10.1007/s00134-012-2536-x

**Published:** 2012-03-30

**Authors:** J. L. Epker, Y. J. de Groot, E. J. O. Kompanje

**Affiliations:** Department of Intensive Care Medicine, Erasmus MC University Medical Centre Rotterdam, P.O. Box 2040, 3000 CA Rotterdam, The Netherlands

**Keywords:** Brain death, Perimortem sperm procurement, Organ donation, Ethics, End-of-life, Proxy consent

## Abstract

**Introduction:**

Although sperm procurement and preservation has been become commonplace in situations in which infertility can be easily foreseen, peri- or postmortem sperm procurement for reproductive use in unexpected coma or death is not generally accepted. There are no laws and regulations for this kind of intervention in all countries and they may also differ from country to country. Intensive care specialists can be confronted with a request for peri- or postmortem sperm procurement, while not being aware of the country-specific provisions.

**Case description:**

A young male patient who suffered 17 L blood loss and half an hour of cardiopulmonary resuscitation was admitted to a university hospital for an ill-understood unstoppable abdominal bleed. After rapid deterioration of the neurological situation, due to severe post-anoxic damage, the decision was made to withdraw life-sustaining treatment. At that moment the partner of the patient asked for perimortem sperm procurement, which was denied, on the basis of the ethical reasoning that consent of the man involved was lacking. Retrospectively the decision was right according to Dutch regulations; however, with more time for elaborate ethical reasoning, the decision outcome, without the awareness of an existing prohibition, also could have been different.

**Conclusions:**

Guidelines and laws for peri- or postmortem sperm procurement differ from country to country, so any intensive care specialist should have knowledge from the latest legislation for this specific subject in his/her country. An overview is provided. A decision based on ethical reasoning may appear satisfying, but can unfortunately be in full contrast with the existing laws.

## Introduction

The first successful retrieval of sperm from a brain-dead patient was reported in 1980 [1]. In 1995 the first semen collection by rectal electro-ejaculation in a brain-dead patient was described and conception from perimortem sperm procurement (PMSP) was brought to the attention of the general public in the UK by Diane Blood [2–4]. Another milestone case was the Parpalaix case in France, where as a result the French Center for the Study and Preservation of Human Sperm petitioned the courts for a full ban on posthumous insemination [5]. In the USA, Gaby Vernoff was the first to conceive with intracytoplasmic sperm injection (ICSI) after the death of her husband [6]. Ever since, there has been increasing worldwide interest in PMSP. Paradoxically, in a recent study of 8 years of PMSP in Israel, in none of the cases in which permission for PMSP was granted was the sperm eventually requested for fertilization use [7].

In other cases, the conclusion was often drawn that reproduction by means of PMSP was, for several different reasons, not ethically justified. In some countries, therefore, laws now prohibit PMSP under all circumstances, whereas other countries designed special laws for these cases, while other countries still lack legal provisions for procedures like PMSP [8].

Intensive care specialists are rarely confronted with this ethical dilemma; accordingly intensive care literature on this subject is scarce. In this article we describe the case of a severe neurologically damaged ICU patient, who was registered as a tissue and organ donor, for whom a request for PMSP was denied.

## Case description

A 30-year-old man was brought to the emergency department of a secondary hospital after a sudden collapse. Ultrasound of the abdomen showed free fluid with the density of fresh blood.

The patient was transferred to the operating room for laparotomy. During surgery, he suffered massive blood loss and a 30-min resuscitation procedure was necessary to regain circulation. After circulation was regained, the patient was transferred to our university hospital. Unfortunately the neurologic situation of the patient deteriorated rapidly on the third day.

The results of the Somato Sensory Evoked Potentials implicated a potentially very bad prognosis and were communicated to the mother of their 2-year-old son, who then asked if it would be possible to procure sperm from her partner to secure the possibility of a second child from this man. After consultation with a clinical ethicist the decision was made not to facilitate sperm procurement. The paramount reason was that written consent of the patient for sperm collection was lacking and consent could not be presumed.

In this phase the physician is required to consult the Dutch donor registry to find out if the patient is registered as an organ and/or tissue donor, which he was. Since the family understood the poor prognosis they agreed with the withdrawal of the mechanical ventilation and supported the wish of the patient to donate his organs and tissues. After circulatory death, both kidneys and the heart valves were used for transplantation.

## Discussion

Although the decision not to proceed with PMSP was legally correct, as gamete harvesting for cryopreservation in both men and women is only justified under Dutch law with a written patient consent, as we learned by analyzing this case, the question is whether the original decision made can also be ethically justified? In this case we do have doubts.

There are six points for discussion:

1. Commonly described reasons for refusal of PMSP

In the past, several cases have been described in which the request for PMSP, or the authorization for the use of the procured sperm, was turned down [9–12]. The reasons given were the lack of proof of an established relationship, a mother or parents who wanted sperm from the dead son, lack of agreement between the relatives of both partners, the deceased patient did not want children when alive, and the lack of a written consent. In our case only the last reason applied.

When a request for PMSP is denied, an often used argument is that the person who should be responsible for the decision never can be certain that the patient would have agreed with it given the circumstances [13, 14]. Therefore in the Netherlands and for example also in the UK gamete procurement in a comatose or perimortem patient is only possible with a signed consent of the patient. The paradoxical outcome of such legislation is, however, that since almost nobody will sign such an advance directive, gamete procurement becomes practically impossible in any unanticipated coma, vegetative state, or (brain) death.

2. The stability of the relationship between the patient and his partner

The patient and his partner had a long-lasting, officially registered relationship with rights that equal those of a married couple in the Netherlands. They were parents of a 2-year-old son and a possible recent miscarriage proved that the family was not regarded as complete yet. Proxies from both sides of the family confirmed the wish for another child in this relationship and they all declared that the man would have agreed with sperm procurement if he had had the possibility to do so, because, as they stated: “It would have been in his line of thinking”.

On the basis of a protocol proposed by Batzer et al. [16] and on a dichotomous key approach for PMSP decision-making, there would have been no reason why PMSP should have been refused in our case [15]. The steps 1, 2, and 4 of this key approach are essential; there is a proven established relationship, there is evidence that the deceased person wanted to have children, and there are witnesses other than the requesting person that can confirm that the deceased person possibly could have agreed with the procedure. In any other case there seems to be no ethical justification for PMSP.

3. A spouse can legally authorize organ procurement and autopsy but not sperm procurement

The most important reason why the clinical ethicist involved in this case advised against sperm procurement was that it may be ethically questionable to assume that a man who wants a complete family still wants it without him being present.

However it seems illogical to us to enable postmortem organ procurement or autopsy without patient consent and at the same time deny the request for sperm procurement.

In the Netherlands proxies are allowed to decide whether or not a patient will become an organ donor if the patient did not leave an advanced directive, or is not registered in the organ donor registry. Likewise proxies are entitled to approve postmortem autopsy. It is important to realize that autopsy is a highly invasive act, harming the bodily integrity, which is in no way serving the interests of the patient. Any kind of tissue can legally be collected during autopsy (even more ethically sensitive tissues like testicular tissue) and stored thereafter and used for research for years, without consent of the patient. These rights are based on the presumption that the proxies do have a reliable idea about the religious, moral, or political thinking of the patient in question and are generally accepted because it facilitates organ donation in individuals who are not registered in a donor registry. Proxy consent for organ donation or autopsy is regarded as altruistic for third parties and in this way serving society or science as a whole. However, when proxies are supposed to be capable of making a “well-judged” decision for a patient concerning organ donation or other postmortem interference with the body, why then do others state that a partner would not be able to make a balanced decision about PMSP? Some argue that the possibility of conflict of interest which would interfere with the proxy’s capacity to provide adequate “substituted judgment” is accordingly much greater than for organ donation. This is, however, not supported by evidence and a conflict of interest is certainly not necessarily present in these situations.

We wonder, which subject will be more discussed within relationships of young couples: organ donation or family planning? PMSP in itself shall never be discussed, but partners will definitely have a reliable idea of how the other partner thinks about reproduction or family planning.

4. Organ donation is considered altruistic, PMSP selfish

Some authors consider asking for PMSP as an act of selfishness, as compared with the altruistic character of organ donation. The presumption that organ donation is without ‘reward’ and therefore not selfish is questionable, as there is an undeniable psychological benefit that is inextricably connected with altruistic actions and “good deeds”. This positive feeling obtained after making a difficult choice is psychologically to be regarded as “reward”.

Furthermore it is assumed that when organ donation is made possible, “society” will benefit. However, it is not society, but a few “lucky” individuals, and often only just one or two, as a result of the disappointing organ quality after circulatory death.

Had PMSP been possible and the partner of our patient had become pregnant, then there were also two individuals that would have taken benefit: the partner that finds hope in new life that is deeply connected with the man she lost and her son that get’s a little baby brother or sister.

5. Timing of sperm procurement

It is generally recognized that procurement before circulatory death is preferred over procurement after circulatory death, because after death the harvesting methods are limited and invasive. Moreover procurement is only successful when performed in the first 24–36 h after death. The patient’s partner asked for the PMSP on the right moment from another point of view (i.e., before withdrawal of life-sustaining treatment, before the official moment of death) because as formulated by White, “when the husband is in a coma or in a persistent vegetative state and they are still married, the wife cannot remarry and cannot have a child legally with another man.… If the husband is dead though, they are not married anymore and the wife is free to marry and legally have children with somebody else, making PMSP not permissible with wife’s consent alone” [17]. On the basis of this point of view, we would have at least had an argument to procure and preserve sperm as was also suggested by the wife of the patient in the case described by Moser [11]. The discussion whether or not it might be used would then follow later as in the “Blood” case [3]. In this perspective it is important to realize that in countries where PMSP is allowed, a 6- to 12-month period for bereavement and reflection is mandatory, before the first attempt for fertilization is initiated.

The fact that the UK High Court, referring to the European law for unimpeded exchange of medical care, made the export of the sperm in the aforementioned “Blood” case possible, potentially provides an escape route for future cases in European countries where PMSP is not allowed or restricted. Retrospectively, we could have brought our patient, before withdrawal of treatment, to Belgium also, to make PMSP possible there.

Unfortunately, there was at that time no overview readily available of the possibilities and regulations in the various European countries. Therefore we analyzed all available literature on PMSP laws and regulations in various countries and summarize these results in Table 1.

**Table 1:** Overview of rules and legislation concerning perimortem sperm procurement and use for fertilization in different countries in and outside Europe [5, 8, 11, 15, 18, 19]

	Prohibited by legislation or guidelines	Written consent obligatory	No written consent obligatory	Not defined in guidelines or legislation
Australia			^a^	+
Belgium			+	+
Canada	+			
Denmark	+			
Estonia	^b^			
Czech Republic		+		
France	+			
Germany	+			
Hungary	+			
Ireland				+
Israel			^a^	+
Italy				+
Japan				+
Latvia				+
Lithuania				+
Malta				+
Netherlands	+			
Norway	+			
Poland				+
Portugal				+
Slovakia				+
Slovenia	+			
Sweden	+			
UK		+		
USA			+	+

^a^Only possible by court order, no special law

^b^Sperm can only be obtained and/or used until a maximum of 1 month after death and only when assisted reproduction was already initiated before death

Although this overview could be of assistance in a case of PMSP request, detailed knowledge of the individual situation is still of the utmost importance; therefore, most doctors in Europe shall consult the juridical department of their hospital for further guidance, when in doubt about the applicability of legislation or lack of clarity of the rules in such a case.

6. The interest of the child to be

A last argument sometimes posted against PMSP is that we are not informed about the potential negative effects on the development of the child to be. Although we do agree that the interest of the child always should be guarded, the fact is that there is no clear evidence available that a child raised in a loving but different system than a mother–father system is less happy, stable, or successful than any other child [20].

## Conclusion

Although a request for PMSP will remain a rare event on the ICU, intensive care specialists should be aware of the practical and legal issues involved, since the decision whether or not to proceed with PMSP can only be taken in a relatively short time-window. Different countries have different laws and regulations in relation to PMSP and each intensive care specialist should have an idea about the country-specific regulations on this subject. Cross-border European medical care may provide a potential escape route for patients in countries where PMSP and/or cryopreservation is not allowed.

The woman in our case had the right and the possibility to give away organs and tissue, to give permission for autopsy, and to become a single mother by insemination of sperm of an anonymous donor, but not the right to become a mother by PMSP from her own legal sexual partner. The question remains whether this is a logical ethical decision or just a flaw in law and reasoning?
